# Managing Late and Long-Term Effects Among Swedish Adolescents and Young Adults Affected by Cancer Following a Wilderness Intervention: Reconnecting with Self and Meaning Through Nature’s Unconditional Presence – A Qualitative Study

**DOI:** 10.1177/10732748251414202

**Published:** 2026-01-20

**Authors:** Heléne Dahlqvist, Ann Ekdahl, Emma Wiklund, Mats Jong, Sveinung Berntsen, Carina Ribe Fernee, Miek C. Jong

**Affiliations:** 1 Department of Health Sciences, Mid Sweden University, Sundsvall, Sweden; 2 Department of Sport Science and Physical Education, University of Agder, Kristiansand, Norway; 3 Research Unit, Sørlandet Hospital, Kristiansand, Norway; 4 Department of Child and Adolescent Mental Health, Sørlandet Hospital, Kristiansand, Norway; 5 The Arctic University of Norway, National Research Center in Complementary and Alternative Medicine (NAFKAM), Department of Community Medicine, Faculty of Health Sciences, UiT, Tromsø, Norway

**Keywords:** adolescents and young adults (AYAs) affected by cancer, cancer survivorship, complementary therapies, health promotion, nature-based interventions, quality of life

## Abstract

**Introduction:**

Adolescents and Young Adults (AYAs) affected by cancer are at risk of experiencing late and long-term effects following cancer diagnosis and treatment. While structured interventions have demonstrated potential benefits for well-being during the intervention itself, little is known about how AYAs affected by cancer engage with and experience nature in their everyday lives beyond the formal program context. The aim of this study was to explore how a selected group of AYAs affected by cancer—who had participated in a nature-based intervention (the WAYA program)—experience nature and its role in supporting and managing late and long-term effects of cancer and its treatment, beyond the context of the program itself.

**Method:**

This qualitative study explored the experiences of nature following the WAYA intervention among AYAs affected by cancer. Data were collected by focus group interviews at a three-month follow-up, using participant-selected photographs as visual prompts to elicit reflection on how nature supports management of late and long-term effects of cancer. A photovoice-inspired approach guided discussion questions and facilitated individual and collective meaning-making. Interviews were audio-recorded, transcribed verbatim, and analyzed using qualitative content analysis.

**Results:**

The analysis of this study showed one theme: Reconnecting with self and meaning through nature’s unconditional presence—beyond human connection. The theme comprises the two categories *Nature as a resource for inner balance* and *Nature as a meaningful companion*, and five sub-categories*: Managing by being mindful, Managing by experiencing rest and being calm, Experiencing metaphoric recognition, Sparking comfort, hope and positive emotions, *and* Nature as a space without obligations.*

**Conclusion:**

This study explored how AYAs affected by cancer engage with nature beyond formal programs, revealing its potential in better management of late and long-term effects. These findings can inform low-threshold, personal meaningful, and sustainable approaches to clinical practice, well-being, and rehabilitation for this vulnerable group.

## Plain Language Summary

Why was the study done?

Adolescents and young adults (AYAs) who have had cancer often experience late and long-term effects from their illness and treatment, such as fatigue, anxiety, and challenges in everyday life. While rehabilitation programs exist, we know less about how AYAs affected by cancer themselves use nature in their daily lives to manage these effects beyond structured programs.

What did the researchers do?

The researchers invited AYAs affected by cancer who had participated in an intervention called the Wilderness program for Adolescents and Young Adults affected by cancer (WAYA) to share their experiences of nature through focus group discussions, photographs, and personal reflections. The researchers analyzed this material to understand how young people describe the role of nature in coping with challenges after cancer.

What did the researchers find?

The study identified one main theme: *Reconnecting with self and meaning through nature’s unconditional presence—beyond human connection*. This theme included two main ways that nature helped:

• Nature as a resource for inner balance – by being mindful and by finding calm and rest.

• Nature as a meaningful companion – by recognizing metaphors in nature, sparking comfort, hope, and positive emotions, and by providing a space free from obligations.

What do the findings mean?

The study shows that nature can play an important role in supporting the well-being of this selected group of AYAs affected by cancer and helping them manage long-term effects in ways that feel personally meaningful and sustainable. These insights suggest that nature could be used as a low-threshold, flexible, and accessible part of rehabilitation and everyday coping strategies for AYAs affected by cancer and potentially for other groups with similar health challenges.

## Introduction

According to the World Health Organization,^1^ cancer is a growing global health challenge because its effects extend beyond the individual’s health, affecting individual economic stability as well as healthcare systems and social development. This makes cancer one of the most common public health issues, with age-standardized incidence rates (ASIR) approximately 790,33 (95% CI: 694.43-893.01) per 100 000 population worldwide.^2^ Due to advances in diagnostics and treatment, survival rates for various cancers have improved significantly over recent decades, although regional differences remain.^3^ The increased survival rate brings a new set of challenges in terms of long-term and late effects related to both the disease and its treatment, highlighting the need for ongoing support and follow-up care.^4^

Among cancer survivors, adolescents and young adults, commonly referred to as AYAs, represent a unique group. The term AYA typically refers to individuals diagnosed with cancer between the ages of 15 and 39^5^. This age span encompasses significant developmental milestones, and a cancer diagnosis during this period can profoundly affect physical, emotional, and social trajectories.^4^ While most AYAs affected by cancer transfer into late survivorship, defined as more than five years since diagnosis, this group often faces a distinct and complex set of challenges because of their diagnosis and treatment. These challenges are usually defined as late and long-term effects,^6^ both of which can significantly affect quality of life.^4,7^

Late effects are defined as symptoms and conditions that can occur months or even years after the treatment has ended.^4,6,8^ AYAs affected by cancer face a wide range of medically confirmed and treatment-related late effects, including cardiovascular disease, endocrine disorders such as thyroid dysfunction and diabetes, neurocognitive impairments, secondary malignancies, sexual dysfunction, fertility issues and disfigurement.^4^ These late effects often require long-term medical follow-up and significantly impact daily functioning, with AYAs affected by cancer reporting lower self-rated physical health, reduced mobility, and diminished participation in work, education, and physical activity compared to their healthy peers.^9^

Long-term effects, on the other hand, refer to health problems or psychosocial difficulties that begin during cancer treatment and persist beyond its completion.^4,6,8^ AYAs affected by cancer are at heightened risk of psychological distress, including anxiety, depression, post-traumatic stress and fatigue.^10^ Fatigue is one of the most frequently reported long-term effects across different diagnostic groups of AYAs affected by cancer and is often described as persistent and limiting, affecting the ability to engage in everyday activities and social life. Fatigue is not only a physical burden but is also frequently reported as a form of mental exhaustion, limiting concentration, motivation and emotional capacity.^10^ In a review of patient-reported outcomes, Tanner et al.^11^ presented several studies that indicated reduced psychological well-being among AYAs affected by cancer. Psychosocial issues are often amplified by the developmental stage they are in, where emotional regulation and coping strategies may not yet be fully developed.^4^ Compared to older populations affected by cancer and healthy peers, AYAs affected by cancer consistently report higher levels of emotional distress and fear of recurrence, which may interfere with everyday functioning including planning for the future.^4,12^

AYAs affected by cancer often report “feeling different” from their peers, missing out on expected life milestones such as becoming more independent or starting their own family.^4^ Employment and financial challenges are frequently reported, with some survivors facing barriers to re-entry into the labor market or educational system.^4^ These disruptions may contribute to a lasting sense of social disconnection and vulnerability.

While survival rates have improved significantly, current care models frequently fall short in addressing the long-term and interconnected challenges experienced by this group.^4^ Unmet mental health needs remain high in young cancer patients and access to age-appropriate psychological care is often lacking.^4^ Current survivorship care models tend to focus on medical surveillance, often at the expense of addressing psychosocial needs and everyday functioning.^4^ Increasingly, researchers and clinicians are calling for flexible, integrative approaches that address both the clinical and everyday realities of cancer survivorship, supporting resilience, autonomy and well-being over time.^4,11^ This highlights the importance of interventions that support mental resilience and help AYAs affected by cancer navigate the emotional aftermath of cancer, underscoring the need for holistic survivorship care.

## The Role of Nature in Health and Recovery

Research shows that nature exposure can affect health, both directly and indirectly and Kuo^13^ identifies several plausible pathways linking contact with nature to improved health outcomes, including enhanced immune function, reduced inflammation and lower levels of stress-related biomarkers. These effects are thought to operate through a combination of environmental conditions and physiological shifts, including increased parasympathetic activity and hormonal balance. Natural settings may also mitigate harmful exposures, such as pollution, heat and noise encountered in urban areas, thereby offering both protective and restorative health benefits. Nature exposure have been shown to be associated with reduced stress and improved mental health^14^ as well as increased physical activity levels (Authors, 2021).^15^

Natural settings are described as non-judgmental, passively supportive and gently stimulating environments that offer opportunities for undemanding activities and spontaneous social interaction.^16-18^ The natural environment also encourages movement, relaxation and multisensory engagement, all of which are associated with improved psychological resilience and quality of life.^19-21^

In the context of severe illness, nature has been found to strengthen self-esteem, autonomy and identity.^16-18^ Previous research has suggested that time spent in nature can support healing processes in individuals with stress-related illnesses^22^ and help maintain a sense of self in conditions such as dementia.^17^ Nature has also been described as a stabilizing and health-promoting resource in the context of severe illness.^23,24^ Nature can function as a catalyst for health-related behaviors, such as physical activity, which in turn contribute to overall well-being.^20,22^ A systematic review and qualitative meta-synthesis by Blaschke^25^ further illustrated how individuals living with or recovering from cancer experienced nature as supportive in various ways, including emotional relief, reconnection with what matters, and opportunities for reflection and healing. Despite many promising findings about nature-based interventions and their effects on health in general, including for AYAs affected by cancer, there are still gaps in the literature. Timko Olson et al.^26^ conclude in their scoping review that many studies have small sample sizes with bias in sampling and that there are few randomized controlled trials.

## The Wilderness Program for Adolescent and Young Adult Cancer Survivors (WAYA-2)

The WAYA-2 study is a randomized controlled trial investigating the effects of a nature-based wilderness intervention for AYAs affected by cancer (Authors, 2022).^27^ Participants were randomly assigned to either a hotel stay (control group) or the WAYA program, which included an eight-day expedition in a natural wilderness setting, followed by a four-day basecamp gathering three months later (Authors, 2023; Authors 2024).^28,29^ The hotel stay included a similarly structured eight-day program at a spa hotel without nature-based activities. The trial has been conducted in Sweden and Norway, and a total of 150 participants were randomized in the study (Authors, 2024).^29^ The present study is embedded in the qualitative research component of the WAYA-2 study and focuses specifically on the Swedish participants’ experiences of nature in everyday life after the eight-day wilderness expedition.

Previous studies have reported that wilderness interventions like the WAYA program increase physical activity and mental and physical well-being of AYA cancer survivors.^30-32^ However, none of these studies applied randomized designs and therefore cannot exclude the possibility that the observed beneficial health effects are caused by factors other than the wilderness program. Furthermore, little is known about how AYAs affected by cancer engage with and experience nature in their everyday lives beyond the formal program context. Understanding these experiences is crucial, as contact with natural environments may offer a unique source of support, restoration, and management that complement medical and psychosocial care. By exploring how AYAs affected by cancer interact with and perceive nature after participating in the WAYA program, this study addresses an important knowledge gap regarding the sustained, everyday role of nature in managing late and long-term effects of cancer and supporting recovery. Such insights may inform the development of accessible, nature-based strategies that enhance long-term well-being for AYAs affected by cancer beyond structured programs.

Therefore, the aim of this study was to explore how a selected group of adolescents and young adults affected by cancer—who had participated in a nature-based intervention (the WAYA program)—experience nature and its role in supporting and managing late and long-term effects of cancer and its treatment, beyond the context of the program itself.

## Methods

### Design

A qualitative approach was chosen to gain insight into participants’ experiences, meaning making and perspectives in relation to nature and its role in supporting the management of late and long-term effects of cancer and its treatment. The study used focus group interviews as the primary source of data. We reported the study following the Consolidated Criteria for Reporting Qualitative Research (COREQ), a 32-item checklist developed by Tong et al,^33^ as detailed in Supplementary Data S1.

As a visual component has been shown to enhance memory, reflection and emotional expression,^34,35^ our study was inspired by the photovoice method. Photovoice is a method often used to document lived experiences, promote dialogue and empower individuals to reflect on strengths and challenges in their environment.^36^ Drawing on Patton,^37^ this design was considered suitable for capturing and exploring the experiences of AYAs affected by cancer and making it more explicit for others to understand.

### Participants and Procedure

**Figure 1.**
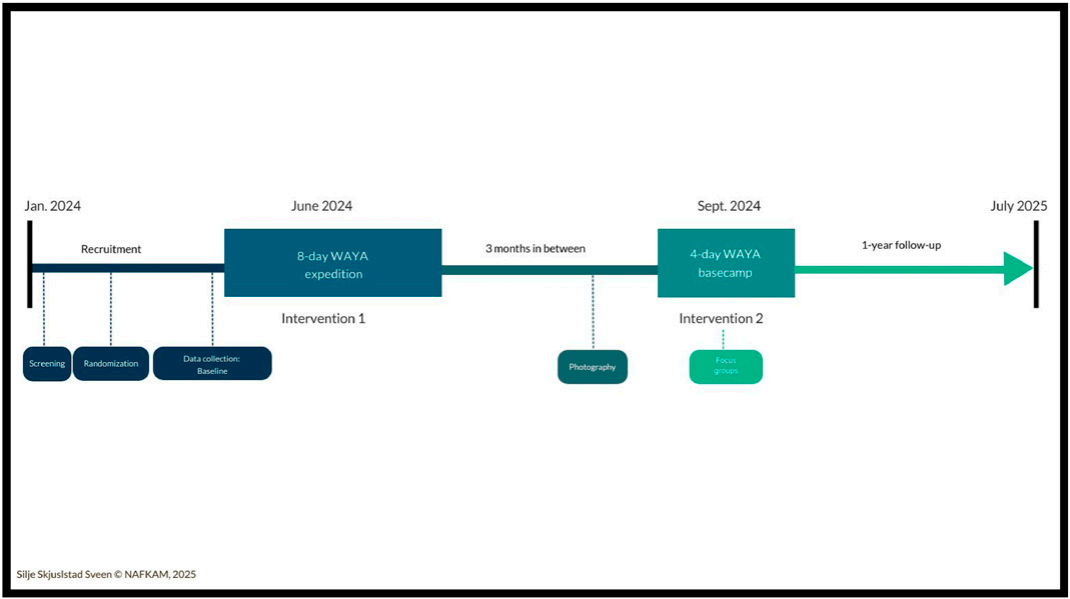
Schematic overview of the WAYA-2 study: Wilderness program for Adolescents and Young Adults (WAYA)

Recruitment of participants into the WAYA-2 study follows a purposive sampling approach directed mainly to those that are members of the Swedish cancer organizations Ung Cancer (Young Cancer) (16-39 years of age) and the Swedish Childhood Cancer Fund, as well as through the social networks of previous participants in the WAYA study (Authors, 2023).^28^ Individuals eligible to participate were AYA’s aged 16-39 years who had received a cancer diagnosis during childhood, adolescence, or young adulthood. No eligibility restrictions were placed on the type of the individuals’ cancer diagnosis or time after cancer treatment. Participants had to be capable of walking at least 2 km without a pause, for which walking aids were allowed. No prior outdoor experience was necessary. Exclusion criteria were ongoing cancer treatment that poses a risk to participation or other medical conditions that will compromise safety. Participants in the current qualitative interview study were contacted about two months after the completion of the eight-day WAYA program intervention. The sample size was estimated according to the concept of information power.^38^ Figure 1 gives a schematic overview of the WAYA-2 study and the timeline of photography and focus group sessions. Information letters (including informed consent) with an invitation to participate in the focus groups were sent by the research team to all (n = 27) Swedish participants in the WAYA program. A total of 18 participants gave their permission and participated in the focus group interviews. The majority (67%, n = 12) had a slight or strong preference for the wilderness program, whereas 11% (n = 2) had a slight or strong preference for the hotel program and 22% (n = 4) had no preference for either. Of the 18 participants only one had previous outdoor experience with backpacking or kayaking. Participants did not receive financial compensation for participation in WAYA-2-study. All necessary outdoor clothing and equipment in addition to meals were made available to participants for participation in the wilderness expedition and basecamp stay. Furthermore, travel costs of participants to and from the wilderness sites were paid for by the study budget. Participation in the WAYA program was therefore without additional costs for participants. Detailed information about the WAYA program and its logistics has previously been published.^39^

### Data Collection

Focus groups and data collection were conducted at the four-day follow-up base camp in September 2024 three months after the first part of the wilderness program in June 2024 (see Figure 1). Participants were asked in advance to take and select photographs that represented their experiences with nature in their daily lives. This was guided by a prompt: How do you experience that nature supports you in dealing with late and long-term effects from your cancer (based on your experiences from the WAYA program or from your own exporiences)? Participants were encouraged to bring 1-2 photos and prepare to share short reflections linked to the photos. The photovoice method informed the structure and preparation of the focus group discussions, including the formulation of guiding questions (SHOWeD).^36^ We adapted the SHOWeD tool to the context of the study and study prompts which resulted in the following questions to guide the focus groups: “What can we see in this picture?”; “How is what is shown in the picture connected to your daily life?”; and “How does that affect your well-being and health?”

Data were collected through three focus group interviews, each consisting of six participants. The photographs, along with participants’ descriptions, served as visual prompts that encouraged discussion and contributed to rich reflections during the focus group interviews which, in line with Krueger et al,^40^ was chosen as the main interview format to encourage interaction, co-reflection and shared meaning-making. The focus groups were conducted in a large lavvu (similar to a tipi tent) raised by the ocean shore, providing a natural and informal setting that aligned with the overall theme of the WAYA-2 study. Each focus group lasted between 100-120 minutes, including a short break. The discussions were led by a facilitator (Authors 1 (female) or 2 (female)), while a third researcher assisted with logistics and took observational field notes (Author 7 (female)). The facilitators were unknown to the participants prior to the interviews, while the assistant reseracher was well known as she was one of the leaders of the 8-day WAYA expedition. During the focus groups, participants presented their photos and accompanying explanations in turn. The rest of the group was invited to respond, reflect, or share related experiences, creating a space for both individual storytelling and collective meaning-making. All focus groups were audio recorded. The recordings were then transcribed verbatim.

To ensure trustworthiness, this study employed the approach of credibility, dependability, confirmability and transferability outlined by Graneheim et al.^41^ AYAs affected by cancer who had all taken part in the WAYA program supported credibility. Their shared background and experiences, in combination with the fact that they knew each other quite well, provided context-rich insights, variation and knowledge about the potential of nature-based interventions for this population.^37,42^ Credibility was further enhanced through transparent documentation of methods, consistent analysis, and collaboration among all researchers.^43^ The study design and data collection were inspired by the photovoice method, a participatory approach that encourages visual storytelling and participant empowerment.^36^ Step three in the photovoice method is community- and participation-based action towards policy and decision makers, grounded in the outcomes of workshops. Although this step was not considered feasible within the current RCT design of the WAYA-2 study, a follow-up project with active AYA participation is planned that includes this third step of the photovoice method.

Dependability was supported through the conduct of the analysis, with the first three authors conducting the analysis of data and all other authors independently reviewing the results. Lastly, all authors discussed the results and reached an agreement. Transferability was supported through detailed descriptions of the WAYA program context, participant characteristics, and data collection, allowing readers to assess relevance to other settings. Presenting results in a theme, categories, and subcategories with illustrative quotations further strengthens confirmability and transferability.^41,43^ Researcher reflexivity and openness to diverse backgrounds helped clarify pre-understandings and enhance the trustworthiness of the study.^41^ A focused sample with detailed and rich dialogue supported by information power justified the limited number of focus groups.^38^

For transparency in this study, the directed nature of the photovoice prompts may have foregrounded positive narratives and limited ambivalent or negative perspectives.^36^ In addition, the prompts were not pilot tested in advance. The self-selection of participants—who had chosen to engage in the WAYA program and this sub-study—may also indicate a predisposition toward viewing nature as beneficial.^37^ While the use of focus groups with prompts and photographs generated rich, emotionally grounded insights, it may have restricted the diversity of perspectives.^34^ These considerations point out methodological limitations and we suggest that future research could include more open-ended approaches to capture a wider range of experiences.^44,45^

### Analysis

Interview texts were analyzed using qualitative content analysis with an inductive approach, following the approach of Graneheim et al.^41^ Initially, the interview texts were read several times to gain an overall understanding, after which meaning units were identified in line with the study’s aim. The meaning units were condensed and coded by Author 3. The codes were then compared to find differences and similarities based on shared patterns in the manifest content into sub-categories and categories (Authors 1, 2, and 3) (Table 1). This phase resulted in a total of five sub-categories and two categories. This process was iterative, going back and forth, comparing codes, categories, and the interview text. In a last step, a theme was identified as a thread of meaning across categories as an underlying latent content.^41^

**Table 1.** Example of the Analysis, Condensed Meaning Units, Sub-categories, Categories, and a Theme

Meaning unit	Condensed meaning unit	Sub-category	Category	Theme
I go out and look at the flowers, what’s growing. What is … You kind of focus on something else that is so clear…. In nature you must not be active but mindful…here you become calm and mindful.	Being in nature makes it easier to relax and to be present. It requires less effort to be mindful	Managing by being mindful	Nature as a resource for inner balance	Reconnecting with self and meaning through nature’s unconditional presence—beyond human connection
When my brain is tired, like, just walking in nature. It is like letting the body do the work while the brain can rest, and I’ll feel calm.	Walking in nature lets my body move while I find rest and calm	Managing by experiencing rest and being calm		
I have two perfect trees where I have a hammock. I’ve looked up and just seen them here. And this but the hope, that just is there suddenly. It comes out of nowhere. You just look up at this. This blue sky, these beautiful trees	Two trees, a hammock, and the sky- hope suddenly appears, in the stillness of that view	Sparking hope and positive emotions	Nature as meaningful companion	
And that’s why it feels so comforting to be out in nature...because there are no demands or obligations there	Nature soothes because it asks nothing of you, it simply lets you be	Nature as a space without obligations		
“I feel a need to almost scream… You walk around and you choke a scream. All I want is just to be able to…. waaah … scream all the frustration, anger, and sadness out. All of it!! The ocean can do that for me…. It screams for me. It does”	I carry a scream inside me frustration, anger, sorrow but the ocean releases it for me. It screams what I cannot	Experiencing metaphoric recognition in nature		

### Ethical Considerations

The responsible body for the WAYA-2 study is Mid Sweden University, and the study was approved by the Swedish Ethical Review Authority (No: 2023-05247-01). The WAYA-2 study was designed in accordance with fundamental ethical principles as outlined in the Declaration of Helsinki^46^ and the guidelines of the Swedish Ethical Review Authority.^47^

All participants received both written and verbal information about the purpose of the study and their rights as research participants. They gave their written informed consent prior to participation. Participation was voluntary, and individuals were informed of their right to withdraw at any time without explanation or consequence. The data were stored on a password-protected server managed by the research team. Before analysis and reporting, all participant details were de-identified, in line with principles for responsible research and data protection.^47^ The focus groups were led by experienced facilitators from Mid Sweden University (Department of Health Sciences). The use of photographs as discussion prompts was introduced with clear instructions to support participant autonomy and ensure emotional safety. In reporting the results, quotes were presented with fictitious names to ensure participants’ anonymity.

### Reflexivity

This study was conducted by a multidisciplinary team with backgrounds in public health, nursing, physiotherapy, sport science, health science, and health services research shaping both design and interpretation. The team’s expertise spans youth mental health, oncology, chronic illness, and participatory methods, offering a broad lens on nature’s role in cancer survivorship.

Authors 1-3 (females) conducted the analyses and their pre-understanding is outlined in the following: Author #1 is a Ph.D. in Public Health Science and contributed experience in youth mental health and participatory methods. Though not directly involved in the WAYA interventions, she interviewed participants from both Wilderness (WAYA-2) and hotel stay (WAYA-1) groups and conducted statistical analyses for WAYA-1. Her external position enabled analytical distance, while her work with vulnerable youth supported a nuanced understanding of psychosocial dimensions. Author # 2 is a Ph.D. in Nursing Science and a registered nurse and brought clinical and research experience with cancer patients and chronic illness. As a logistic facilitator in the WAYA-2 intervention, Author #2 had direct insight into participants’ lived experiences. She also conducted interviews in the current study and her qualitative research background supported a nuanced interpretation of emotional and embodied aspects. Author #3 is a MPH and licensed physiotherapist and contributed clinical knowledge of long-term conditions and participated as a facilitator in the Wilderness intervention. Her practical experience helped contextualize nature’s role in physical and psychological adaptation.

The authors acknowledge that their professional and personal interest in nature-based interventions may have shaped initial assumptions—particularly the belief that nature can support healing and emotional regulation. This is also the main reason why athors 4-7 (author 4-5 males; author 6-7 females), did not take lead on the anayses as they are the PIs of the WAYA-2 in Sweden and Norway respectively and have a profound professional and personal interest in nature-based interventions. To mitigate potential bias, the team engaged in ongoing reflexive dialogue and remained attentive to diverse participant narratives. The combination of embedded and external researcher roles supported a balanced interpretation, enhancing the study’s credibility and depth.

## Results

The study included 18 participants. An overview of participant characteristics is presented in Table 2.

**Table 2.** Participant Demographics, Diagnoses, Time since Completion of Cancer Treatment, and Late and Long-Term Effects

Variables	Response categories	Number of participants (Total n = 18)
Age min-max, md		24-39, 34
Sex	Women	15
	Men	3
Education	High school	1
	College/University	13
	Other	4
Civil status	Married/Registered partner	8
	Unmarried	10
Housing situation	Living by themselves	6
	Living with immediate family	8
	Living with partner	4
Ethnicity	One/both parents born in Sweden	14
	Both parents born outside Sweden	4
Employment status	Full time working	9
	Part time working	2
	Full time education	1
	Unable	5
	Unemployed	1
Cancer categories	Blood and bone marrow cancers	3
	Solid tumors	15
Time since last treatment	≤3 months	4
	4-11 months	2
	1-5 years	10
	>5 years	2
Late and long-term effects	Fatigue	7
	Anxiety	7
	Pain	7
	Mild neurocognitive disorders	6
	Depression	5
	Stress	4
	Mental health problems	4
	Sleeping problems	4
	Impaired mobility	2
	Other*	14

*Other late and long-term effects included: neuropathy, concentration-, balance-, climacteric-, intestinal-, and connective tissue problems, migraine, burnout, auditory sensitivity, diplopia, nystagmus, eczema, heart palpitations, peroneal nerve paralysis, and tinnitus.

The analysis of this study resulted in one theme: *Reconnecting with self and meaning through nature’s unconditional presence—beyond human connection.* The theme comprises two categories and five sub-categories and captures how nature was experienced by AYAs affected by cancer in supporting and managing late and long-term effects of cancer and its treatment (see Table 3). The following sections present the results organized by the theme, categories, and subcategories, along with illustrative quotes and images from participants.

**Table 3.** Overview of Subcategories, Categories, and the Theme

Sub-category	Category	Theme
Managing by being mindful	Nature as a resource for inner balance	Reconnecting with self and meaning through nature’s unconditional presence—beyond human connection
Managing by experiencing rest and being calm		
Experiencing metaphoric recognition	Nature as meaningful companion	
Sparking comfort, hope and positive emotions		
Nature as a space without obligations		

### Nature as a Resource for Inner Balance

#### Managing by Being Mindful

Participants described that living with late and long-term effects of cancer meant experiencing feelings of anxiety, stress, or inner restlessness. However, participants described an increased sense of mindfulness while immersed in natural environments. Mindfulness was described as a process of anchoring oneself in a broader existential framework — one that transcends interpersonal relationships and encompasses a deeper sense of meaning. This connection to nature was described as supporting them in managing their challenges of balancing hope as well as hopelessness. This was described by participants when engaging in activities that required their attention by observing nature. The participants articulated observing nature using all their senses, such as watching how the light shifted through the branches of a tree, listening to the sound of leaves rustling in the wind, feeling the stability of a block of stones, tasting fresh berries, or smelling soft soil, all of which drew their attention and distracted them from overwhelming emotions and mental strain. Further, they noted that mindfulness supported their ability to manage and better recognize their physical and emotional needs, such as reducing stress. Being in nature, invited to focus on details, actively and gently pulled them along with nature’s own pace. Furthermore, the participants described how anxious thoughts that constantly swirled in their minds eased while they were in nature, allowing them to relax. One participant explained the importance of nature as a resource for inner balance and mindfulness:

*“… I go out and look at the flowers, what's growing. What is ... You kind of focus on something else that is so clear…. here you must not be active but mindful…here you become calm and mindful”* Susanne

**Figure 2.**
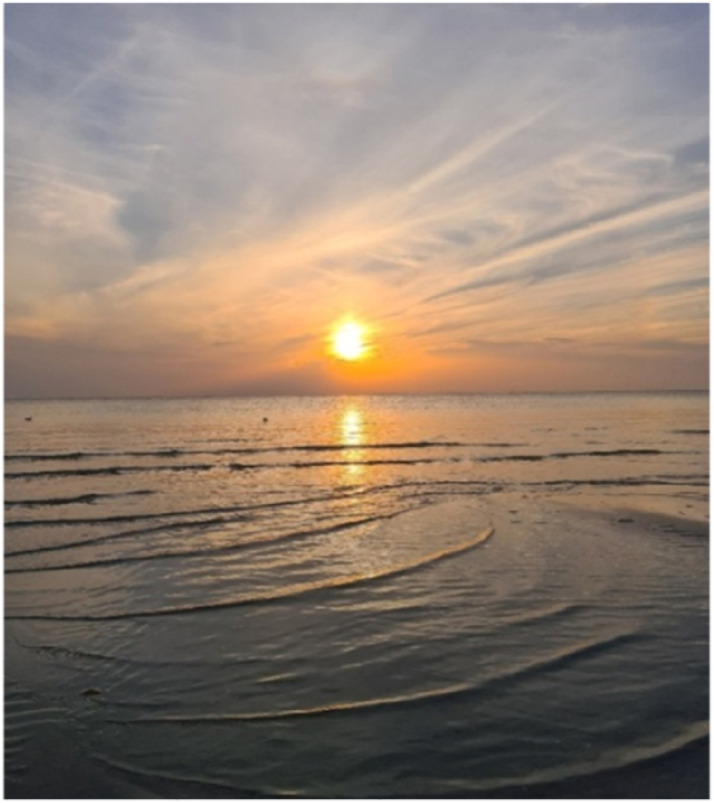
Picture A: “Warmth, serenity, calmness, often get the feeling that things will work out when I watch a peaceful sunset.” Vera

One of the other participants expressed how anxious thoughts would go away as nature appeared to promise that things would work out, which is exemplified in the photo (Figure 2) and caption of a beautiful sunset:

#### Managing by Experiencing Rest and Being Calm

Participants described that late and long-term effects of cancer meant living with fatigue, exhaustion and reduced cognitive function and mental fatigue. To rest in nature was described as mentally restorative and effective in alleviating both physical and psychological fatigue. The participants described that rest in nature involved finding a pause, peace, and a place where the mind could rest and a moment of stillness with few external distractions. Additionally, participants described the rhythms inherent in natural processes as supportive in managing the late and long-term effects of cancer. Spending time in nature was a pause in a bubble outside of their everyday life and a place to slow down, calm down, turn inward, gather their thoughts, and choose what to focus on. Some participants emphasized the importance of rest with an accelerated everyday life, noting that the stark contrast rendered rest especially salient and offered perspectives and meaning that transcended human connection, giving a deeper bond and sense of relationship.

**Figure 3.**
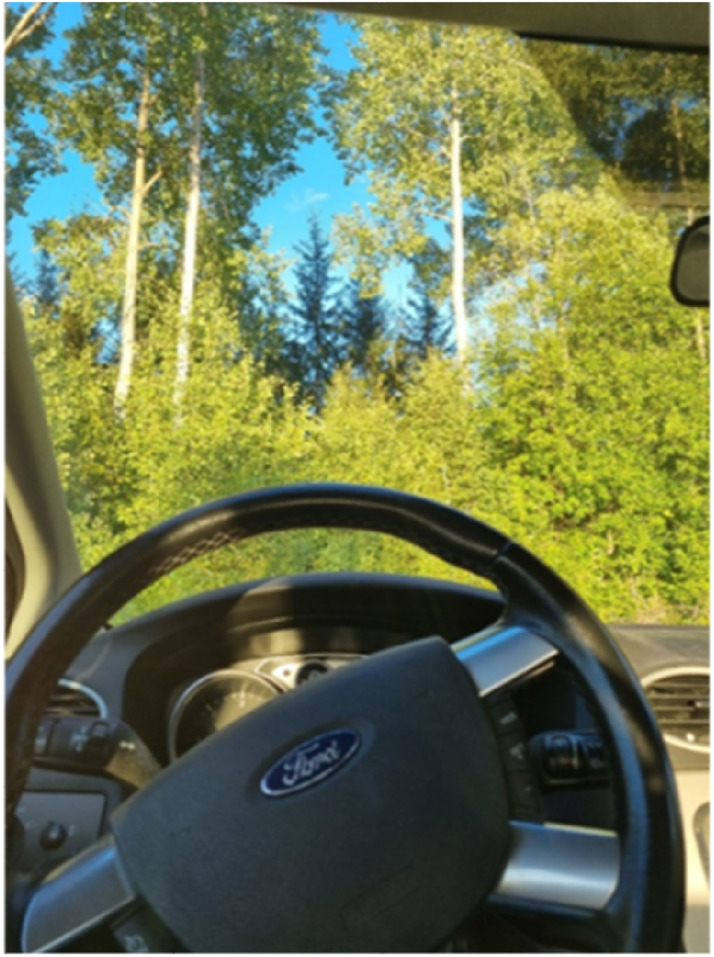
Picture B: “I take little breaks in nature to recharge. Here I stopped the car on the way home from work and meditated and looked at nature. The brain gets to rest when I look at nature.” Stella

The experience of rest in nature — characterized by mental and sensory stillness — was described as facilitating bodily ease and readiness for physical activity. In addition, energy that would normally be consumed by mental strain could be directed toward physical activity and help them increase and regain physical capacity and a sense of renewed strength, both physically and emotionally. Participants described that by resting in nature, the emphasis was on relaxation, making the specific activity less significant, surrounded by calm and non-demanding impressions. Moreover, the experience of rest in nature entailed a process of deceleration — pausing activities, withdrawing from external pressures, and engaging in reflection on past experiences. Stella recounted the numerous times she had taken small breaks on her way home from work (Figure 3):

Participants described that being in nature was spreading a sense of calm and stillness through impressions. These sensations were associated with being in more remote environments, such as a summerhouse in the archipelago. Spending time in nature was described as an opportunity to reconnect with themselves, sometimes just for a moment, and sometimes for longer periods. In everyday life, participants described just taking a short break by going into the garden or sitting in the car looking at nature could make a difference and bring stillness. One participant explained the importance of rest in nature:

*“When my brain is tired like, just walking in nature…letting the body do the work while the brain can rest, and I’ll feel calm”* Nora

### Nature as a Meaningful Companion

#### Experiencing Metaphoric Recognition

Participants described trees, mountains, and natural landscapes as reminders of something sustainable that remains over time, not easily moved or changed. Being in nature was described as emotional safety, a sense of protection, and a calm and caring space that strengthens and steadies. Participants described how being in nature made them sense recognition for the changes they had gone through, both physically and emotionally due to late and long-term effects of cancer. They recognized themselves in aspects of nature reflecting participants’ inner or outer changes in what was broken, endured, adapting to abnormalities, mirroring their internal emotional states, or standing out as physically different. Likewise, participants described experiencing a metaphoric recognition of change, struggle, difference, and endurance while also highlighting nature’s role as an unwavering, nonjudgmental and meaningful companion. This was described as mirroring their human experience with illness. The unpredictability of their health mirrored that of the natural world; as their bodies fought to heal, participants recognized parallels in nature’s adaptive forces — such as rivers carving new paths when obstructed — and reflected on their own capacity to adjust to their ongoing challenges. Being in nature spoke directly to their inner experience and offered quiet recognition, not as a cure, but as a companion. One participant described metaphoric recognition in nature as a support:

*I feel a need to almost scream… You walk around* [in everyday life] *and you choke a scream. All I want is just to be able to .... waaah...scream all the frustration, anger, and sadness out. All of it!! The ocean can do that for me…. It screams for me. It does*. Vera

**Figure 4.**
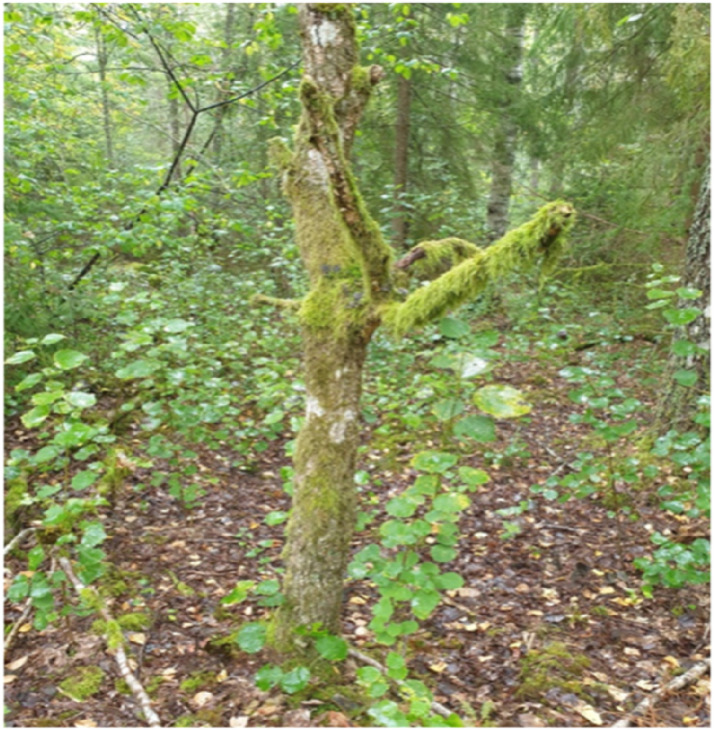
Picture C: ”I feel affinity with nature. It gives me comfort and energy. This mirrors us humans. Here is “me” with a secure trunk, moss that has grown for a long time in some parts, some branches have been cut, the surroundings are young, delicate and light. I often feel like the moss tree. In a bubble of my own outside the ordinary world.” Stella

Another participant recognized herself in a battered, moss-covered tree (Figure 4):

#### Sparking Comfort, Hope and Positive Emotions

Participants described that living with late and long-term effects of cancer entailed a sense of loss. This included the loss of abilities, plans, dreams and aspects of identity, but also the loss of others affected by cancer. The participants described being in nature as offering comfort for their losses, noting the warmth of the sun on their skin, the quietness, and the sense that time was limitless. Observing butterflies or birds was experienced as a gentle, affirmative reminder of deceased loved ones, providing a symbolic space that invited the presence of grief and facilitated emotional processing. Participants reflected on nature’s cyclical patterns as reminders that renewal is always possible — a perspective deeply intertwined with their experiences of loss, restoration, and relational connection. Hence, participants described that the need for comfort is often intertwined with the need to be seen in one’s suffering. They needed to express their emotions without filtering them, something they often felt that they had to do in the presence of friends and family. This created a need for hope and positive energy, and being in nature functioned as a source of feelings of joy, evoking positive emotions and playfulness. The participants described nature as a supportive unconditional source of hope in navigating the late and long-term effects of cancer, offering reconnection with self and meaning that extended beyond human connection. This helped them make sense of illness, mortality, and transformation. Nature was also described as serving as a place to long for. Positive emotions were described by the participants as feelings of upliftment and inspiration. This counterbalanced getting stuck in rumination. One participant described:

*“I have two perfect trees where I have a hammock. I look up, and then hope is there suddenly. It comes out of nowhere…I just look up at this blue sky, these beautiful trees just like he was....”* Selina

**Figure 5.**
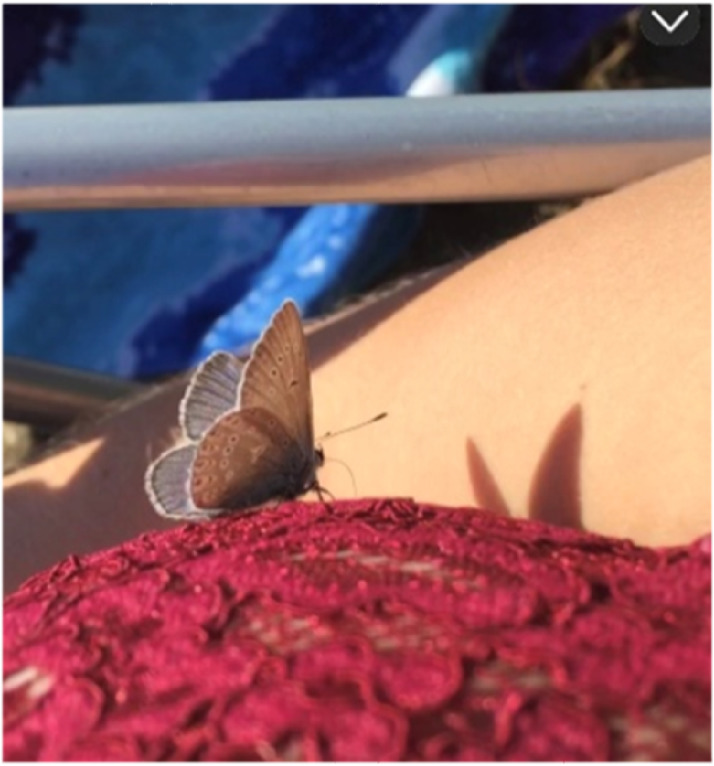
Picture D: “The butterfly reminded me of my deceased friend coming to visit…it gave me comfort, and my eyes teared up” Erika

One participant captured a butterfly in a photograph when it unexpectedly landed on her (Figure 5). She described interpreting the event as personally meaningful, evoking a sense of comfort and positive emotion:

#### Nature as a Space Without Obligations

Participants described reflective experiences in nature as fostering greater emotional awareness and attune to their inner states. Nature was further perceived as a safe, non-judgmental space free from demands that enabled the acknowledgment and release of emotions and their associated feelings. Participants felt being offered refuge in nature managing long-term effects such as overwhelming emotions, stress, anxiety and fatigue. Taking a break from everyday demands and spending time in nature was described by participants as a meaningful aspect of daily life, fostering a sense of temporal abundance and spaciousness. Participants described being in nature as offering space to let go and simply be—without the need to perform, explain, or conceal—providing time to reorient their perspective on life after cancer and fostering a renewed appreciation for life. Consequently, nature was described as a space without obligations, offering an elevated sense of vitality, connection, freedom, renewal, and the creation of meaning. Furthermore, strong support came from being surrounded by the natural world, a space where life can simply be experienced rather than managed. Participants described being in nature as an experience beyond human connection, offering support in their ongoing efforts to manage the late effects of cancer and its treatment. One participant said:

*“And that’s why it feels so comforting to be out in nature...because there are no demands or obligations there”* Astrid

**Figure 6.**
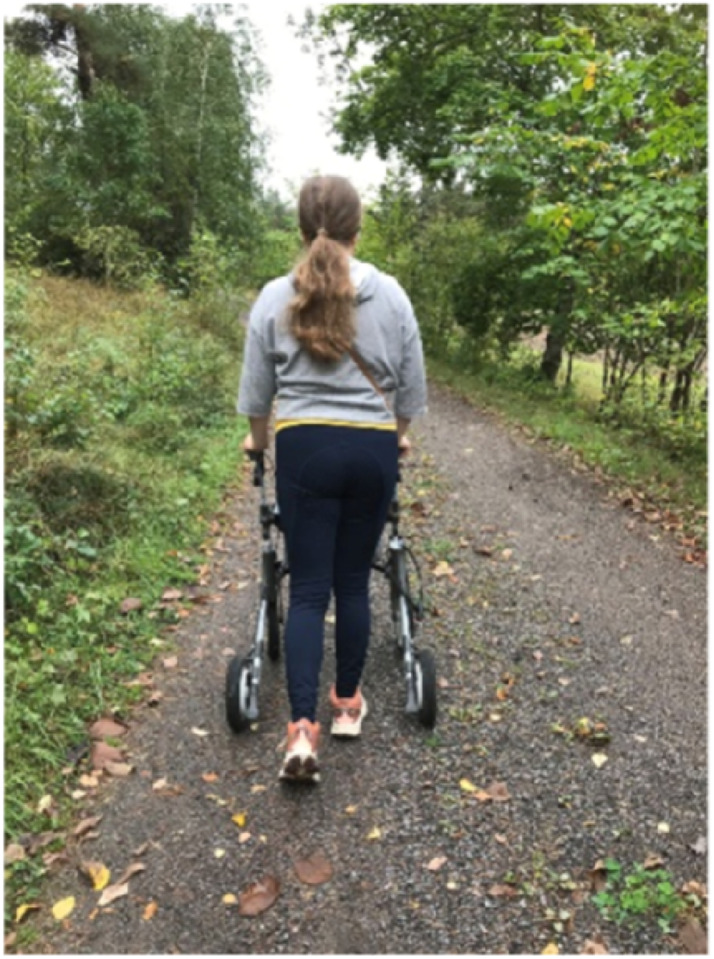
Picture E: “Activities in nature strengthen my physical, mental, and psychological well-being…I feel capable with a sense of freedom from bodily limitations…it feels like an unconditional source, nature” Anette

Nature was also described as offering a sense of freedom from bodily limitations, enabling participants to experience competence and capability. Anette illustrates this in a picture that may be paradoxical at first, since the use of a walker on a dirt road may be challenging (Figure 6).

## Discussion

This study aimed to explore how a selected group of adolescents and young adults affected by cancer—who had participated in a nature-based intervention (the WAYA program)—experience nature and its role in supporting and managing late and long-term effects of cancer and its treatment, beyond the context of the program itself.

The main finding in this study is captured in the theme Reconnecting with self and meaning through nature’s unconditional presence—beyond human connection. This study contributes novel findings but also aligns with previous research. A novel finding is the understanding of the importance of experiencing rest and restoration in nature. The WAYA program may impact managing late and long-term effects of cancer and its treatment, by the possibility of facilitating rest in nature, as expressed by participants. This resulted in both readiness for physical activity and renewed strength, and a pause from their everyday life. For AYAs affected by cancer, who often report cognitive fatigue and impaired executive functioning,^4^ being in nature appears especially valuable^48^ as it is crucial to find a suitable rest rhythm to maintain their health. Mental imagery of rest and non-rest can help people reflect on their need for rest within their life context. Preserving energy by resting implies a sense of harmony among people with different kinds of long-term illnesses. When linked to nature and health, these concepts offer valuable insights into care and support processes.^49^ In contrast to the emphasis on rest, Pálsdóttir^22^ and Blaschke^50^ highlighted nature-based activities as energizing and revitalizing. Further, nature may contribute to eudaemonic well-being and support the resilience and quality of life of AYAs affected by cancer.^20,21,51^

Participants in the present study expressed how being in nature with all their senses helped them shift focus or capture their attention. This offered a distraction from mental strain and difficult emotions and helped the participants to become more aware of their physical and emotional needs. They described a shift from cognitive overload to mindful, embodied presence, as such representing a movement toward present-moment awareness. As shown in previous studies^4,52^ this need has been expressed by AYAs affected by cancer who commonly experience internal restlessness, worry, or difficulty slowing down.

Participants expressed how being in nature provided metaphoric recognition, mirroring the unpredictability of their health, emotional states, personal, physical, and adaptive changes, and offering a space for emotional expression. Natural elements, such as heavy sea waves, symbolized complex emotional states. These encounters with nature were expressed by participants as affirming and validating. The study’s results contribute to an understanding of nature as a meaningful companion, and therefore an important source of support. This is in line with previous research on how nature helps support identity making and emotional regulation in a variety of populations such as young people with mental health issues in urban settings^16^ and people living with dementia.^17^ Similarly, Blaschke et al.^50^ described how individuals undergoing treatment recognized themselves mirrored in natural elements. However, in their study, such recognition of nature’s cycles sometimes evoked discomfort by reminding individuals of mortality and vulnerability. This divergence may partly reflect the differing participant contexts: Whereas Blaschke et al.^50^ included individuals in active treatment or with terminal prognoses, the current study focused on AYAs affected by cancer living with late and long-term effects, whose perspectives may be shaped by a greater psychological distance from acute illness.

The study’s results show how nature helped the participants to reframe personal concerns, accept change, and reconnect with something larger than themselves. Frumkin et al.^53^ further emphasize that contact with nature can support existential well-being by providing a place that invites consolation and reflection, without being in a religious context which is also in line with Blaschke et al.^50^

Participants expressed that nature was a space of emotional safety and stability. Being in nature often comes with quiet expectations due to personal preferences and memories. This aligns with findings by Birch et al.^16^ and Owen et al.,^17^ who highlight nature’s role as a non-judging and flexible support. Frumkin et al.^53^ add that low-complexity environments reduce cognitive load and support emotional balance. Nature doesn’t promise anything, its stillness and unpredictability invite letting go of control, a place to let go of expectations, sensory overload, and obligations.

In this sense, participants’ expressions of connecting an apophatic understanding with nature invited an encounter with the world marked by humility and an acceptance that the most profound truths may not be spoken but simply sensed. This perspective aligns closely with Næss’^54^ concept of the *ecological self*, which emphasizes the interconnectedness of humans and the natural world. The category *Nature as a resource for inner balance*, encompassing the subcategories *Managing by being mindful,* and *Managing by experiencing rest and being calm*, reflects the idea that engagement with nature supports a widened sense of self. According to Næss,^54^ the ecological self includes not only one’s personal identity but also an awareness of and identification with the natural environment. Mindfulness and the restorative qualities of nature can thus be understood as ways in which AYAs affected by cancer reconnect with this broader self, finding equilibrium and a sense of calm that transcends conventional coping strategies.

Similarly, the category *Nature as meaningful companion* with subcategories including *Experiencing metaphoric recognition*, *Sparking comfort, hope and positive emotions*, and *Nature as a space without obligations*, illustrates Næss’s^54^ notion that nature is not merely a backdrop for human activity but a relational entity with intrinsic value. Through metaphoric recognition and emotional engagement, participants formed meaningful relationships with the natural world, which could provide support independently of human social interactions. This resonates with Næss’s^54^ ecological philosophy, where the self is extended into the natural environment, allowing nature to function as a companion, source of hope, and facilitator of existential well-being.

Taken together, the findings suggest that nature provides both restorative and relational dimensions of support for AYAs affected by cancer, consistent with Naess’s^54^ view that well-being arises not only from human-to-human interactions but also from profound, identity-affirming relationships with the more-than-human world.

## Strengths and Weaknesses

The strength of this study is the rich interview data from the participating AYAs affected by cancer. In striving for authenticity, the authors have attempted to describe the participants’ experiences in ways that enable the reader to see nuances and variations, and virtually hear their voices.^55^ The rich data material was suitable for descriptions of the participants’ everyday lives, their subjective world, and of the sustained everyday role of nature in managing long-term effects of cancer and supporting recovery being in nature. This study shows their possibilities for nature as an unconditional source of support in managing late and long-term effects to well-being and rehabilitation for this group outside of, or following, structured programs.

This study has some weaknesses. The second, third, fourth and seventh authors were facilitators in the WAYA program and established a relationship with the participants. However, the combination of embedded and external researcher roles in the analysis supported a balanced interpretation, enhancing the study’s credibility and depth. Given the self-selection of participants who had chosen to engage in the WAYA-2 study and the fact that most participants in this sub-study preferred the wilderness program, the selected nature of this sample is considered a limitation. Results of this study are therefore not generalizable to the general AYA cancer population and mainly restricted to the experiences of a smaller group of AYAs affected by cancer in Sweden.

## Implications

The global surge in demand for mental health services has prompted researchers and practitioners to explore readily available and holistic group treatments, including nature-based interventions. Rather than offering a fixed model, the findings of the current study can offer a mapping of the various ways in which nature may support AYAs affected by cancer in managing late and long-term effects. These insights may guide young people in exploring supportive strategies in their daily lives and serve as a resource for clinicians seeking to better understand how nature can be integrated into psychosocial support and health-promoting practices. The findings demonstrate that nature can fulfill a variety of supportive functions tailored to individual needs and preferences, particularly for AYAs affected by cancer who often face complex and persistent late or long-term symptoms. This highlights nature’s potential as a flexible and meaningful component of personalized survivorship care. Rather than being a passive backdrop, nature can be seen as an active enabler for improving health. Zerbe et al.^56^ states that health benefits depend on active engagement with nature’s components, characteristics and dynamics such as intentionally engaging multiple senses i.e., touching plants, smelling soil, listening to birds, tasting herbs and observing seasonal and environmental dynamics.

Furthermore, the findings highlight the potential of nature as an accessible and flexible resource in cancer survivorship care and rehabilitation, but also for other groups with similar symptoms such as anxiety and chronic fatigue.^57^ Positive impacts connected to nature’s potential have been reported in previous research to increase neuro-cognitive function, wellbeing, and overall mental health as well as to reduce symptoms, perceived levels of stress, and emotional states.^57,58^ By recognizing the multiple functions that contact with nature may fulfil—ranging from emotional regulation and cognitive restoration to opportunities for agency—health care providers are afforded a broader framework for tailoring support to the heterogeneity of needs among AYAs affected by cancer. This understanding extends beyond traditional biomedical or psychosocial interventions, suggesting that nature-based approaches may function as a complementary component in individualized care and rehabilitation strategies. As pointed out by Truant et al.,^59^ cancer care systems often prioritize biomedicine, standardized protocols, and evidence-based treatments, resulting in rigid structures that limit individualized, person-centered care and overlook group-specific needs. As health systems confront the growing complexity posed by increasing numbers of cancer survivors, recognizing these structural and attitudinal barriers is vital to advancing quality and equity. Drawing on Truant et al.^59^ who argue that by amplifying the voices of survivors, the findings of the current study invite a shift beyond conventional metrics toward a deeper understanding of the lived experiences shaped by cancer.

At the policy level, the study underscores the relevance of ensuring equitable access to restorative natural environments as part of public health and survivorship infrastructures.^60^ Policies that integrate nature-based approaches into cancer rehabilitation programs, community health initiatives, or broader sustainability agendas may serve to enhance long-term quality of life for AYAs affected by cancer as well as other groups experiencing comparable symptoms, such as anxiety or chronic fatigue.^61^ In this respect, the findings may inform strategic planning and resource allocation, highlighting nature not merely as a setting for health promotion but as an active, flexible, and cost-effective element of supportive care.

## Further Research

Although the proportion of funded studies examining structural and social determinants—as well as mechanisms driving disparities in health care and outcomes among cancer survivors—has increased over time, critical gaps remain.^62^ AYAs affected by cancer are particularly underrepresented in survivorship research, and so are studies addressing psychosocial support and long-term quality of life.^62^ Nature-based interventions are emerging as promising yet still underexplored approaches for enhancing well-being among AYAs affected by cancer. Future research should prioritize these gaps by conducting large scale RCTs focusing on innovative, holistic interventions that include nature as a supportive resource.

A logical next step of the current study would be to conduct a longitudinal study following the same group of participants over time, to examine how their use of nature as a supportive resource evolves. This approach would allow for the exploration of both delayed and long-term effects, as also emphasized by Janssen et al.^4^ This could provide insight into what conditions are needed to sustain nature-based coping strategies in the long term and how AYAs affected by cancer can be supported in maintaining access to meaningful nature experiences beyond the intervention itself. In addition, implementations research is another area that should be a priority, e.g., the practice of prescribing nature as a health-promoting intervention within the health care system^63^ for AYA’s affected by cancer remains under-researched and would benefit from further exploration.^60^ Another area for research is to study a broader group of AYAs affected by cancer and possible facilitators and barriers for them to engage with nature. Some evidence shows lack of knowledge, information, and awareness regarding follow-up care of healthcare professionals and survivors.^64^ Further, urgent priorities include theory‑driven research, development, testing, and implementation of multidisciplinary models specifically designed for AYAs affected by cancer.^65^

Completing the third step of the photovoice methodology, reaching policy makers and stakeholders, is another area of interest. By presenting participants’ photos with descriptive texts, policymakers and stakeholders can gain a deeper understanding of how access to and spending time in nature can support and improve health.

## Conclusions

Adolescents and Young Adults (AYAs) affected by cancer who participated in the WAYA program experienced reconnecting with self and meaning through nature’s unconditional presence—beyond human connection. Our study illustrates how this selected group of AYAs affected by cancer engage with and experience nature in their everyday lives beyond the context of a formal wilderness program. Such experiences offer a valuable complement to conventional medical, psychosocial, and rehabilitation efforts. By examining how AYAs affected by cancer interact with and perceive nature after participating in the WAYA program, the study addresses an important knowledge gap concerning the sustained, everyday role of nature in managing long-term effects of cancer and supporting recovery. These insights may guide the development of low-threshold, personally meaningful, and sustainable approaches to well-being and rehabilitation for this group outside of, or following, structured programs.

## Supplemental Material

Supplemental Material - Managing Late and Long-Term Effects Among Swedish Adolescents and Young Adults Affected by Cancer Following a Wilderness Intervention: Reconnecting with Self and Meaning Through Nature’s Unconditional Presence – A Qualitative Study Supplemental Material for Managing Late and Long-Term Effects Among Swedish Adolescents and Young Adults Affected by Cancer Following a Wilderness Intervention: Reconnecting with Self and Meaning Through Nature’s Unconditional Presence – A Qualitative Study by Heléne Dahlqvist, Ann Ekdahl, EmmaWi klund, Mats Jong, Sveinung Berntsen, Carina Ribe Fernee, Miek C. Jong in Cancer Control

## Data Availability

Data from this study is available on request from the corresponding author.*

## Appendix

### Abbreviations

AYA: Adolescents and Young Adults
SHOWeD: See Happening Our lives Why Do
WAYA: The Wilderness Program for Adolescent and Young Adult Cancer Survivors
